# Effect of Short-Term Ageing Treatment on Bending Force Behavior of Commercial Nickel-Titanium Archwire

**DOI:** 10.3390/ma16031008

**Published:** 2023-01-21

**Authors:** Asad Munir, Muhammad Fauzinizam Razali, Muhammad Hafiz Hassan, Gérald Franz

**Affiliations:** 1 School of Mechanical Engineering, Universiti Sains Malaysia, Nibong Tebal 14300, Pulau Pinang, Malaysia; 2 Laboratoire des Technologies Innovantes, UR UPJV 3899, Avenue des Facultés, Le Bailly, 80025 Amiens, France

**Keywords:** NiTi archwire, superelastic, bending behavior, short-term ageing treatment

## Abstract

Superelastic nickel–titanium (NiTi) archwires have become the preferred archwire for orthodontic alignment and the levelling stage due to their ability to exert a light force on teeth throughout a wide range of tooth movement. The magnitude and trend of the force exerted on the malposed tooth is influenced by the orthodontist’s consideration of the size and geometry of the NiTi archwire during orthodontic therapy. In this work, a novel approach of a short-term ageing treatment was utilized to modify the magnitude and trend of the bending force of a commercial superelastic NiTi archwire. The bending behavior of the superelastic NiTi archwire was altered by subjecting it to different temperatures in an ageing treatment for 15 min. The bending behavior of the aged NiTi archwire was examined using a three-point and three-bracket setup. The commercial NiTi archwire’s bending forces in both the three-point and three-bracket configurations were successfully altered by the 15 min ageing treatment. During unloading in the three-bracket arrangement, the NiTi archwires aged at 490 °C or 520 °C exhibited a lower magnitude and more consistent force compared to the NiTi archwires aged at 400 °C or 430 °C. Ageing the archwire for 15 min at 490 °C produced a suitable size of Ni_4_Ti_3_ precipitate, which makes the wire more flexible during bending and reduces the unloading force in the three-bracket bending configuration. The short-term aged NiTi archwire could be used to enhance the force delivery trend to the malposed tooth by lowering the amplitude of the force delivered and sustaining that force throughout the orthodontic treatment duration.

## 1. Introduction

Fixed appliance treatment is a method used by orthodontists to straighten teeth with orthodontic brackets and archwires. In fixed appliance therapy, the archwire plays a crucial role in correcting tooth position. When bent in the bracket system, the archwire’s spring back property exerts an external force on the malposed tooth. It has been established that the force delivered by the archwire to the malposed tooth should be mild and continuous, in the range of 2.0 N or less, to reduce patient discomfort and prevent irreversible damage to the periodontal tissue [1,2]. In comparison to stainless steel archwires, superelastic NiTi archwires exert a low and steady force while bending, making them the wire of choice for most fixed appliance treatments [3]. Archwires made of beta titanium and stainless steel are typically used for space closure and detailing, while superelastic NiTi archwires are commonly employed for levelling and aligning. In addition, owing to its reversible martensitic phase change, this superelastic characteristic enables the archwire to recover its original straight arch shape when the applied force is withdrawn.

Several test models, including cantilever bending [4], three-point bending [5,6,7], and the three-bracket model [3,8], have been utilized to understand the flexural behavior of NiTi archwires to date. It is claimed that superelastic NiTi archwires exhibit a constant bending force behavior during the loading and unloading cycle, and this constant force trend can be observed when the archwires are bent in a cantilever and three-point arrangement. Unfortunately, as the NiTi archwires recover from a considerable deflection of 4.0 mm or more in the bracket system, the continuous unloading force changes into a negative gradient force trend [9,10]. This negative gradient force trend indicates that the amount of force applied to the teeth keeps increasing as the tooth travels to a new position, which is not conducive to achieving a healthy tooth movement rate. The experimental study described in [11] revealed that the unloading force rose from 1.0 N to 4.8 N when the bracket displacement decreased from 3.3 mm to 0.75 mm.

It is believed that the transition of bending force of NiTi archwires from constant to gradient behavior in orthodontic bracket design is associated with the sliding friction components. As the bent archwires slide within the bracket slot to restore their original straight shape, the archwire presses against the bracket’s corner surfaces, thus generating sliding friction. Previous work in [11] indicated that the unloading curve is changed to a lower force level due to the resistance to the archwire’s sliding motion provided by the friction at the wire-bracket interface. The negative gradient force trend during unloading was produced by a lessening of this sliding friction as archwire deflection in the bracket system decreased [10]. This claim was supported by the results of the archwire sliding test conducted by the authors in [12,13,14], who found that the NiTi archwire created higher friction when considering a greater bending deflection. These studies also observed that larger archwire sizes resulted in higher sliding friction, implying that the archwire presses the bracket corner harder when sliding.

Ageing treatment is the most frequent hardening method used to improve the thermal and mechanical properties of Ni-rich NiTi alloys. This treatment method improves the superelastic properties of aged NiTi alloy by introducing a Ni-rich precipitate, a secondary phase, into the matrix of the alloy. It is well established that the size and distribution of precipitates in NiTi alloys change with ageing temperature and time [15,16]. Ageing a NiTi material between 300 °C and 600 °C for 30 min dynamically alters the thermal transformation temperature and the transformation stress [17,18]. It is suggested that deforming NiTi alloys causes the Ni_4_Ti_3_ precipitate to generate local shear stress inside the alloy matrix, hence reducing the critical stress required for martensitic transformation [19]. Until now, research on the impact of ageing on NiTi alloy was limited to tensile deformation, where a significant decrease in tensile stress of 300 MPa was detected when the ageing temperature was increased from 300 °C to 400 °C [20].

To the best of our knowledge, no reports of applying a short-term ageing treatment to commercial superelastic NiTi archwires to improve their current flexural behavior can be found in the literature, regardless of the suggested ageing conditions. This work aims to determine if a short-term ageing treatment may be used to modify the force–deflection behavior of superelastic NiTi archwires. A commercial NiTi archwire was subjected to a short-term ageing treatment at varying temperatures. A three-point and three-bracket setup was utilized to assess the variations in bending force of aged NiTi archwires over loading and unloading cycles. The proper choice of ageing temperature for short ageing therapy may aid in lowering the gradient force behavior of superelastic NiTi archwires in the bracket system, hence improving the force delivery trend to the teeth during the orthodontic treatment.

## 2. Materials and Methods

In this investigation, a commercial superelastic NiTi orthodontic archwire (Masel) with a 406.4 µm diameter was utilized. The archwire’s atomic composition was Ti-50.8 at.% Ni, as determined by EDS analysis. The microstructural and bending specimen were prepared by cutting the straight section of the NiTi archwire into a 10 mm and 50 mm long specimen. The specimens were then solution treated for 15 min at 920 °C in a sealed quartz glass tube furnace (GSL-1100X) with a flow of argon gas, before being quenched in cold water. This method of solution treatment is essential for eliminating the effects of a previous heat treatment. A titanium getter powder was also inserted into the heating tube during the heat treatment to prevent the specimen from oxidizing at high temperatures. The ageing process was carried out at temperatures ranging from 370 °C to 520 °C, with a temperature increment of 30 °C. Each specimen underwent the ageing process for a continuous duration of 15 min. At the completion of the ageing process, the specimens were quenched in cold water to prevent unwanted low-temperature reactions.

Microstructural analysis samples were prepared by mounting 10 mm-long aged specimens on epoxy resin and hardener, then grinding and polishing them using a MetPol^®^ 2 V machine. Starting with 300 grit, the surface of the specimen was grinded to 400, 600, 800, and 2000 grit before being polished with 3 to 0.05 μm diamond suspensions on a DiaMAt polishing cloth using Meta DiTM as a lubricant. Compressed air was used to dry the specimens after they were washed in distilled water and then removed with isopropyl alcohol. Following that, the samples were etched for 15 s in Kroll’s reagent (1–3 mL HF, 2–6 mL HNO, 100 mL water). A Zeiss Supra 35 field emission scanning electron microscope was used for the microstructural analysis (SEM). The SEM was set to 15 kV acceleration voltage and 200 pA probe current.

**Figure 1:**
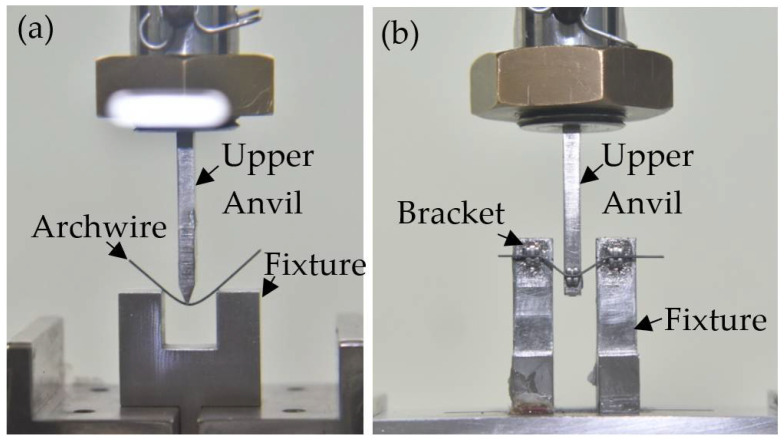
NiTi specimen under bending using (**a**) three-point bending jig and (**b**) modified three-bracket bending jig.

A three-point bending test was carried out to assess the force–deflection behavior of aged NiTi archwires. This test was conducted on a three-point bending jig as seen in Figure 1a, which is also used for a four-point bend test in determination of bond quality in diffusion bonded titanium alloy parts [21,22]. The three-point bending jig was manufactured in compliance with ISO 15841: Dentistry—Wires for use in orthodontics [23], an international standard. In accordance with the specification, the indenter and support point ends were set to have a radius of 0.1 mm, and the distance between these two supports was set to 10.0 mm. The three-point bending test was performed using an Instron 3367 universal testing machine equipped with a 500 N load cell. The NiTi specimen was deflected to 3.1 mm and then allowed to restore to its original straight form by retracting the indenter at a crosshead speed of 1.0 mm/min. The test was conducted at 26 °C ambient temperature. Three repetitions of the bending test were conducted, each time with a separate specimen to ensure force–deflection consistency. During the test, the Instron Bluehill™ material testing software (Norwood, MA) tracked and recorded the force–deflection data.

A modified three-bracket bending jig was used to examine the force–deflection behavior of an aged NiTi specimen in a bracket system. This jig was created by substituting an orthodontic bracket for the indenter and sharp fixture, as seen in Figure 1b. Three stainless-steel (grade 316L) orthodontic brackets, each with 0.46 mm slot height and 2.80 mm slot width, were employed. Multiple studies [8,24,25] have considered this three-bracket design because it approximates the well-known problem in levelling produced by the significant displacement of the canine tooth against the lateral incisor and the first premolar tooth. The two-support fixture was adjusted so that the space between the brackets was 7.5 mm, corresponding to the typical distance between maxillary teeth [10]. The NiTi specimen was inserted into the slots of the three brackets to begin the bending test. The NiTi specimen was bent to a deflection of 4.0 mm by advancing the indenter downward at a rate of 1.0 mm/min, and then allowed to restore to its original straight form by withdrawing the indenter at the same rate. To assure uniformity, the bending test was conducted three times with a different specimen in each run. The Instron Bluehill™ material testing software (Norwood, MA, USA) displayed and recorded the force–deflection measurements during the test.

## 3. Results

**Figure 2:**
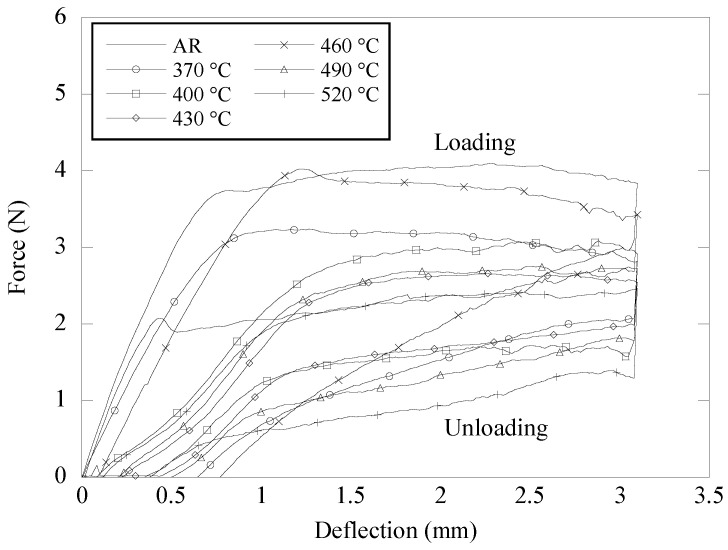
Force–deflection curves of aged NiTi archwires obtained from the three-point bending test.

Figure 2 depicts the force–deflection curves of aged NiTi archwires when subjected to a three-point bending test. The creation of a force plateau during the loading and unloading cycles demonstrates that each archwire was deformed due to superelastic behavior. The loading cycle began with a brief linear slope of bending force up to a deflection of 0.5 mm, reflecting the bending stiffness of the NiTi archwire. The force plateau recorded throughout the loading cycle is indicative of the deformation of the NiTi archwire resulting from the stress-induced martensitic transformation. In the meanwhile, the lower force plateau recorded during the unloading cycle indicates the reversal of the archwire phase transformation when the bending load is removed.

Overall, the as-received NiTi archwire demonstrated the greatest loading and unloading force at all deflections. During the loading cycle, the as-received (AR) archwire registered a maximum loading force of 3.8 N at a deflection of 3.1 mm. The unloading cycle began with a dramatic reduction in force, and from a deflection of 2.5 mm to 0.5 mm, the force eventually stabilized between 2.0 N and 2.2 N. It is worth noting that the bending force decreased as soon as the ageing treatment was applied to the as-received NiTi archwire. For instance, after ageing the NiTi archwire at 520 °C, the unloading force at 2.0 mm deflection dropped from 2.4 N to 0.9 N. This significant decrease in unloading force magnitude indicates a decrease in the bending stiffness of the aged NiTi archwire.

**Figure 3:**
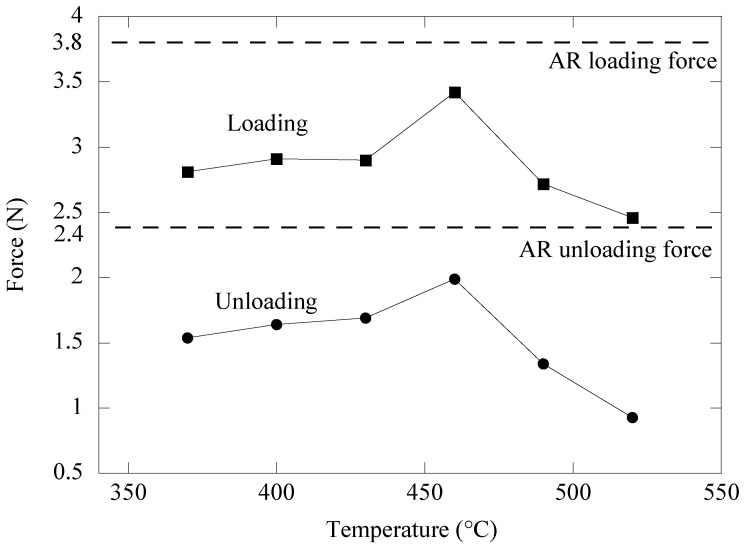
Variation in loading and unloading forces of aged NiTi archwires obtained from the three-point bending test.

Figure 3 depicts the change in loading and unloading forces of superelastic NiTi archwires after 15 min of ageing at various temperatures. The magnitude of the forces was taken from Figure 2. According to ISO 15841: Dentistry—Wires for use in Orthodontics [23], the loading force was measured at a deflection of 3.1 mm, whereas the unloading force was measured at a deflection of 2.0 mm. Overall, it is clear that the short-term ageing treatment successfully affected the bending behavior of the NiTi archwire by lowering the loading and unloading forces below those of the as-received archwire. The loading and unloading force of the as-received archwires were 3.8 N and 2.4 N, respectively. It can be shown that raising the ageing temperature from 370 °C to 460 °C increased the loading force and unloading force of the NiTi archwire from 2.8 N to 3.4 N and from 1.5 N to 2.0 N, respectively. However, as the ageing temperature progressed to 490 °C and 520 °C, both bending forces began to show a decreasing trend. It was discovered that the NiTi archwire aged at 520 °C for 15 min seemed to have the lowest loading and unloading force of 2.5 N and 0.9 N, respectively. These low loading and unloading forces indicate that among the aged specimens, the specimen aged at 520 °C had the lowest flexural stiffness.

**Figure 4:**
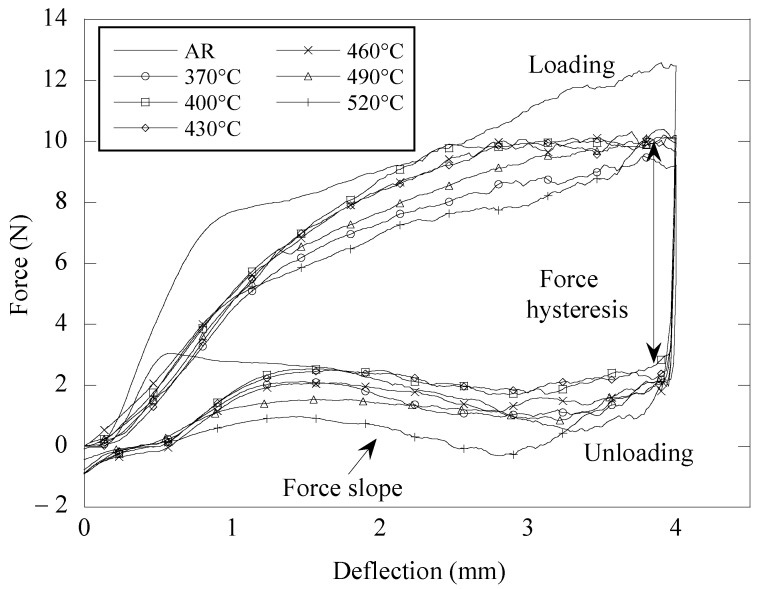
Force–deflection curves of aged NiTi archwires obtained from the modified three-bracket bending test.

Figure 4 depicts the force–deflection curves of the aged NiTi archwire in the three-bracket bending arrangement. When comparing the force–deflection curve in Figure 4 to the one in Figure 2, it is clear that the NiTi archwire has undergone a transition from a constant force trend to a gradient force trend. The loading cycle of the NiTi archwires began with the formation of a linear force slope at a minor deflection of around 0.9 mm, followed by the formation of a moderate force slope as the bending deflection progressed to 4.0 mm. The unloading cycle was initiated by a rapid decrease in force levels until deflection reached 3.5 mm. As the amount of deflection decreased from 3.5 mm to 1.5 mm during unloading, a noticeable force slope was also identified. This force slope exhibited by the superelastic NiTi archwire when bent in the bracket system was attributed to the variation in sliding friction as the archwire pushed the bracket corners with varying intensity along the deflection path [26]. Even though there are two force curves on the force–deflection graph, the unloading force represents the spring back force utilized to move teeth during orthodontic treatment and is thus the focus of this study.

Additionally, the flexural behavior of the NiTi archwire in the three-bracket system was effectively adjusted by ageing treatment by making the archwire more flexible. This is demonstrated by the significant reduction and increase in loading and unloading forces as the as-received NiTi archwire was exposed to ageing procedures. For instance, at a deflection of 4.0 mm, the loading force of the as-received NiTi archwire decreased from 12.5 N to 9.9 N after the ageing treatment at 520 °C was taken into account. This adjustment to the bending behavior of NiTi archwires decreased the hysteresis between the loading and unloading forces. In comparison, the as-received NiTi archwire exhibited a force hysteresis of up to 11.0 N at 3.0 mm deflection; however, this hysteresis value was drastically decreased to 8.3 N when the archwire was aged at 490 °C.

In addition, the archwire aged at 520 °C revealed an unloading force of −0.3 N at a deflection of 2.8 mm. The negative magnitude of the force signifies the end of the sliding motion, as the spring-back force of the archwire is no longer sufficient to overcome the excessive friction at the archwire–bracket contact surfaces. As a result, the sliding movement of the archwire was immediately halted. While the middle bracket was designed to return to its initial position at the end of the unloading cycle, the negative force magnitude is the minimum force magnitude required to overcome the sliding friction and allow the archwire to move again for the remaining deflection. This negative force behavior prior to the unloading of the NiTi archwire from the vertical bracket is comparable to what Naziris et al. [27] observed in an earlier study.

**Figure 5:**
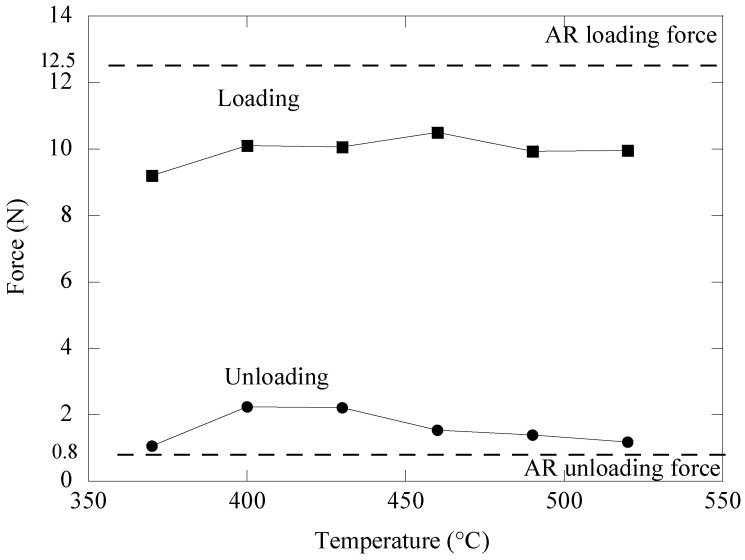
Variation in loading and unloading forces of aged NiTi archwires obtained from the modified three-bracket bending test.

Figure 5 illustrates the change in loading and unloading forces exhibited by the aged NiTi archwires when bent in the three-bracket arrangement. The bending forces were acquired from Figure 4, where the forces were measured with a deflection of 4.0 mm during loading and 3.5 mm during unloading. For comparison, the dashed lines show the loading and unloading force magnitude of the as received NiTi archwire. The flexural stiffness of the NiTi archwire bent in the bracket configuration has clearly been affected by the ageing treatment. This is demonstrated by the substantial decrease in loading force magnitude between the as-received archwire and the aged archwires. For instance, at 4.0 mm deflection, the loading force of the archwire decreased dramatically from 12.5 N to 9.9 N when aged at 520 °C.

Furthermore, the ageing temperature has the impact of increasing the loading force from 9.2 N to 10.5 N as the archwires were aged from 370 °C to 460 °C. Meanwhile, when the ageing temperature was increased to 490 °C and 520 °C, the loading force began to show a force decrease trend to around 9.9 N. The NiTi archwires aged at temperatures between 370 °C and 490 °C exhibited larger unloading force magnitude than the as-received NiTi archwire. A maximum unloading force of 2.2 N was found in the NiTi archwire aged at 400 °C. As the ageing temperature was raised from 430 °C to 520 °C, the unloading force of the NiTi archwire steadily decreased. The archwire aged at 370 °C seemed to have the lowest unloading force of 1.07 N when compared to all other aged archwires. Heat treating the NiTi archwire between 400 °C and 430 °C would be useless because it would increase the unloading force to more than 2.0 N, which would be too high to induce optimal tooth movement.

**Figure 6:**
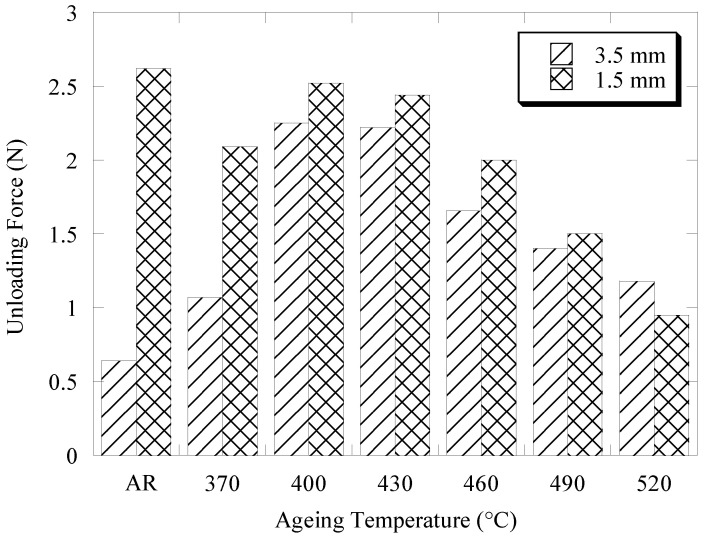
Variation in unloading force exhibited by the as-received and aged NiTi archwires at 3.5 mm and 1.5 mm deflection in the modified three-bracket bending setup.

Figure 6 depicts the unloading forces exhibited by the as-received and aged NiTi archwires in the three-bracket bending arrangement at 3.5 mm and 1.5 mm deflection. Overall, it was found that the NiTi archwire consistently displayed a lower unloading force at 1.5 mm when compared to the 3.5 mm deflection, with the exception of the archwire aged at 520 °C. The force difference between these two deflections indicates that force was exhibited by the NiTi archwire in a negative slope manner during the unloading cycle. The as-received archwire demonstrated the greatest unloading force difference of 2.0 N, with the unloading forces of 0.6 N and 2.6 N at 3.5 mm and 1.5 mm deflection, respectively. High intensity friction experienced by the archwire during sliding motion is believed to be responsible for this large force difference, as it decreases the unloading force of the archwire to a smaller magnitude prior to the unloading cycle. As the friction intensity decreases at lower deflection magnitudes, the unloading force of the archwire recovers to its initial magnitude at higher force levels, hence producing a negative force slope on the force–deflection curve (see Figure 4). On the other hand, it was seen that the ageing treatment effectively reduced the force difference, from as much as 1.0 N for the archwire aged at 370 °C to 0.1 N for the archwire aged at 490 °C. This discovery indicates that the archwire aged at 490 °C exhibits almost a constant unloading force of about 1.4 N to 1.5 N as the bracket displacement decreases during the unloading cycle.

**Figure 7:**
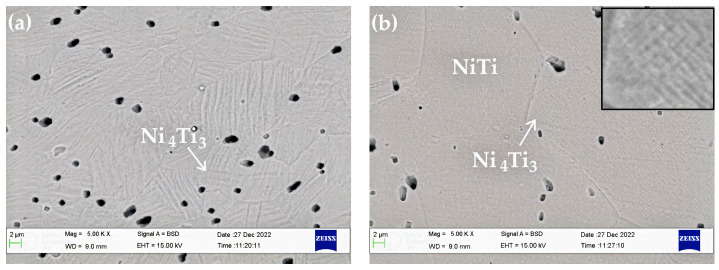

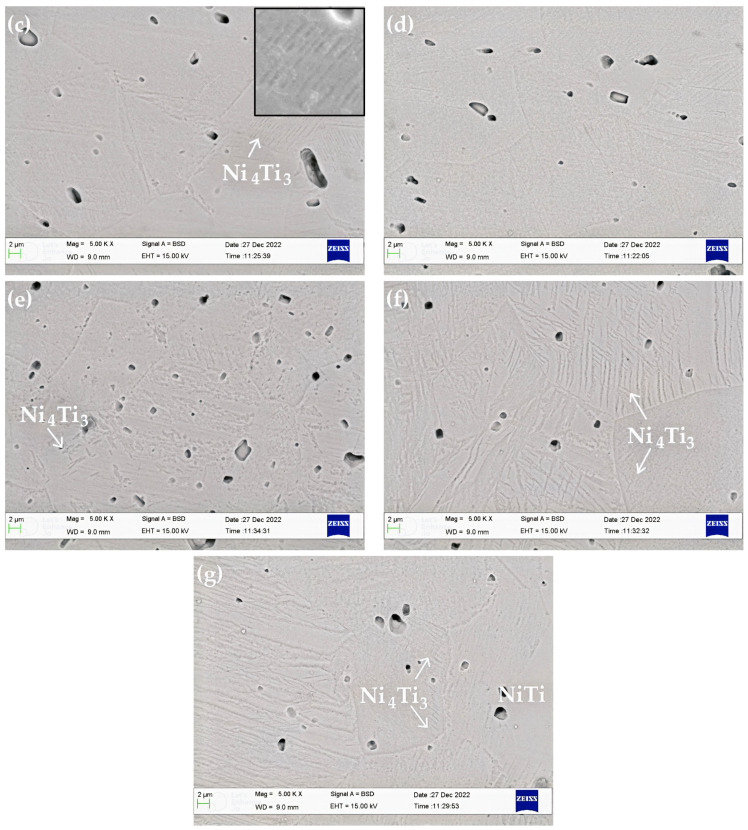
SEM images of NiTi archwires: (**a**) AR archwire, (**b**) aged at 370 °C, (**c**) aged at 400 °C, (**d**) aged at 430 °C, (**e**) aged at 460 °C, (**f**) aged at 490 °C, and (**g**) aged at 520 °C.

Figure 7 depicts the microstructures of the as-received and aged NiTi archwires. Overall, the microstructure of the NiTi specimens is composed of an austenite matrix phase and a lenticular-shaped Ni_4_Ti_3_ precipitate. The lenticular disk-like morphology of the Ni_4_Ti_3_ precipitate agrees with what has been found in previous research [28,29]. Different sizes and orientations of the Ni_4_Ti_3_ precipitate were generated when the NiTi specimen was aged at various temperatures. Figure 7b,c clearly shows that the specimens aged at low temperatures, such as 370 °C and 400 °C, have almost the same microstructure, and the Ni_4_Ti_3_ precipitate is barely visible in the SEM image. The insets in these two figures show a magnified view of the Ni_4_Ti_3_ precipitate, which is estimated to be around 1 μm in length. Meanwhile, the size and appearance of this Ni_4_Ti_3_ precipitate became clearly apparent in Figure 7f,g, as their length increased to about 4 μm when the NiTi specimen was aged at high temperatures of 490 °C and 520 °C. Furthermore, when comparing the microstructures of the as-received and aged specimens, the precipitate distribution was slightly denser and the grain size was slightly smaller with the as-received specimen. This might be owing to differences in ageing temperature and duration employed during the manufacture of the as-received archwires.

## 4. Discussion

The aim of this study was to explore the applicability of short-term ageing treatments in altering the bending behavior of commercial superelastic NiTi archwires. The ageing treatment was performed at different temperatures for 15 min. This heat treatment procedure was introduced to resolve the gradient force trend issues that commercial superelastic NiTi archwires display when bent in the orthodontic bracket system. This paper proposes an appropriate short-term ageing treatment condition, which could help to reduce the rate of force changes as the archwire unloads from large deflections. This mechanical behavior modification of the commercial NiTi archwire is critical in order for the archwire to exert a lower and consistent force on teeth throughout the orthodontic treatment duration.

The current study’s findings and discussion demonstrate the bending behavior of a 0.4 mm NiTi archwire following a 15 min heat treatment. The findings presented here offer an early indication of the applicability of a short ageing treatment time in modifying the bending force magnitude of the commercial archwire in three-point and three-bracket configurations. In this study, the three-point bending test was used to determine the effectiveness of a short-term ageing treatment in altering the flexural properties of the as-received archwire. The test was carried out in accordance with the technique and settings outlined in ISO 15841: Dentistry—Wires for use in Orthodontics [23]. This standard enables users to analyze the flexural behavior of orthodontic archwires with the least potential influence from sliding friction. This is due to the archwire being allowed to freely slide over the surface of the two supports during the bending procedure. However, when an orthodontic bracket is used, the bend generated by the archwire at the bracket slot prevents the archwire from easily sliding at high bracket displacement.

The ageing treatment strategy was adopted in this study owing to its efficiency in changing the tensile deformation behavior of NiTi shape memory alloys as described in earlier research [30,31,32,33]. Figure 3 and Figure 5 demonstrate that ageing treatment can be utilized to adjust the magnitudes of the loading and unloading forces of the as-received NiTi archwire in both three-point and three-bracket configurations. The unloading force of the aged NiTi archwires was consistently lower than the unloading force of the as-received archwire in the three-point bending arrangement. This observation indicates that the aged NiTi archwires are more flexible than the as-received archwire. The variation in loading and unloading forces of the aged NiTi archwire corresponds to the differences in precipitate size inside the archwire matrix, as shown in Figure 7b,g. Ni_4_Ti_3_ precipitates with a length of 4 μm produced in the matrix of a NiTi specimen aged at 490 °C are believed to be suitable for decreasing loading and unloading bending forces. Meanwhile, the friction between the archwire and bracket surfaces is expected to decrease in the bracket arrangement as the ageing NiTi becomes more flexible under stress. As a result, the sliding friction is less likely to postpone the unloading force to a smaller magnitude, hence most of the unloading forces of the aged archwires are higher than the as-received archwire, as shown in Figure 5.

Previous studies have shown that the precipitation process following ageing treatment plays an important role in changing the hardness, compression, and tension behavior of Ni-rich NiTi shape memory alloys [34,35]. The nucleation of Ni_4_Ti_3_ precipitates is claimed to provide a strong surrounding local stress field inside the austenite matrix of the NiTi alloy, lowering the critical stress required for martensitic transformation. In this work, the bending force from the three-point bending setup changes in proportion to the size and orientation of the precipitates, with smaller bending forces measured when specimen nucleated larger Ni_4_Ti_3_ precipitates at high temperatures of 490 °C and 520 °C. The smaller bending force indicates that the transformation of the parent austenite phase of the aged NiTi archwires to a martensite phase occurred at a lower stress level during bending.

The original idea of employing NiTi archwire during orthodontic treatment to give consistent force to the tooth may no longer be valid in the situation of bending the archwire in the bracket arrangement. The loading and unloading forces obtained from the three-bracket bending setup, for example, do not indicate a comparable force plateau as produced in the three-point bending (see Figure 2). When deformed in the bracket arrangement, the as-received and aged NiTi archwires exhibited a positive and negative slope trend in the loading and unloading force curves, respectively. These force slope curves were generated as a result of the change in sliding friction experienced by the NiTi archwire at high deflection. To date, it is understood that friction increases the size of the archwire force during loading and delays the force during unloading. The dependence of the archwire force slope trend on sliding friction has previously been quantitatively investigated by modifying the coefficient of friction at the wire–bracket contact interface. The force slope of a 0.4 × 0.56 mm NiTi archwire was reported to vary at a rate of −0.46 N/mm for incremental increases in the friction coefficient (0.1) [11]. Therefore, it is essential to tune the flexural behavior of the NiTi archwire to prevent it from producing excessive force during the treatment duration, hence creating patient discomfort. It has been documented that a 0.46 × 0.64 mm NiTi archwire in an orthodontic bracket arrangement can exert a force of up to 44.0 N at 3.0 mm deflection [36].

There was a clear correlation between the flexural stiffness of the archwire and the force variation created when the archwire was unloaded in the bracket arrangement. Based on Figure 3 and Figure 6, it was observed that the NiTi archwire with lower flexural stiffness exhibited less variation in unloading force as it recovered from a 4.0 mm deflection in the bracket configuration. For instance, the archwire aged at 490 °C, which exhibited a lower unloading force of 1.3 N in a three-point bending configuration, demonstrated the least force fluctuation of 0.1 N as it recovered from a deflection of 3.5 mm to 1.5 mm. In contrast, the as-received archwire, with the greatest unloading force of 2.4 N in three-point bending, exhibited a significant force variation of 2.0 N. This discovery implies that the NiTi archwire with higher bending stiffness caused more friction during sliding because it imparted the bracket surface with more force, delaying the unloading force to a greater extent. During orthodontic treatment, a substantial fluctuation in force magnitude delivered by the NiTi archwire to the malposed tooth should be avoided, since the ideal tooth movement rate is known to be 1.0 mm/month under a light and consistent force of between 0.10 N and 2.0 N [37]. This is due to the fact that the production and removal of bone cells as a result of tooth movement is strongly reliant on the amount of force applied to the tooth. Frequent changes in the periodontal ligament stress should be avoided, since they will disrupt the present cellular responses of tooth movement [37].

A flexible superelastic NiTi archwire is highly preferred in the early stages of orthodontic treatment because it can be easily secured by the orthodontist into the bracket slot during the installation procedure. According to the findings of this study, a more flexible NiTi archwire could deliver adequate and consistent force to the teeth during archwire deflection recovery. The adjustment of the NiTi archwire force through ageing treatment must be done carefully to ensure the archwire force’s sustainability and appropriateness to induce tooth movement. For example, an archwire made of NiTi should not be aged at a temperature of 520 °C for 15 min, as this might cause the archwire to become too pliable and cause it to lose its spring back capacity over friction when recovering from a large deflection. When this happens, the archwire can no longer slide freely within the bracket slot, which impedes further tooth movement. As an alternative, the ideal temperature for an aged NiTi archwire is 490 °C, as it promotes a steady and constant force of about 1.4 N to 1.5 N during unloading from 3.5 mm to 1.5 mm deflection. Taking into account these ageing conditions may help orthodontists and manufacturers enhance the force delivery of commercial NiTi archwire, which will directly benefit the dental digital workflow [38]. The current findings also imply that ageing treatment may be used to control the mechanical deformation of NiTi implants or retainers, hence resolving problems with oral discomfort [39] and masticatory system disturbances [40].

## 5. Conclusions

The present study investigated the bending behavior of a commercial superelastic NiTi archwire that had been aged for 15 min at various temperatures. The following conclusions can be deduced from this study:

1. A 15 min ageing treatment successfully modified the bending forces of the commercial NiTi archwire under both the three-point and three-bracket configurations.
2. The NiTi archwires aged at 490 °C to 520 °C demonstrated a lower magnitude and more consistent force during unloading in the three-bracket configuration than the NiTi archwires aged at 400 °C to 430 °C;
3. Ni_4_Ti_3_ precipitate produced after 15 min of ageing at 490 °C, with an average size of 4 μm, makes the archwire more flexible while bending, resulting in a decreased unloading force in the three-bracket bending configuration.

## Data Availability

Not applicable.

## References

[1] Krishnan V., Davidovitch Z. (2006). Cellular, molecular, and tissue-level reactions to orthodontic force. Am. J. Orthod. Dentofac. Orthop..

[2] Noda K., Arai C., Nakamura Y. (2010). Root resorption after experimental tooth movement using superelastic forces in the rat. Eur. J. Orthod..

[3] Alobeid A., Dirk C., Reimann S., El-Bialy T., Jäger A., Bourauel C. (2017). Mechanical properties of different esthetic and conventional orthodontic wires in bending tests. J. Orofac. Orthop..

[4] Bilinska M., Kristensen K.D., Dalstra M. (2022). Cantilevers: Multi-Tool in Orthodontic Treatment. Dent. J..

[5] Nespoli A., Villa E., Bergo L., Rizzacasa A., Passaretti F. (2015). DSC and three-point bending test for the study of the thermo-mechanical history of NiTi and NiTi-based orthodontic archwires. J. Therm. Anal. Calorim..

[6] Shahmir H., Nili-Ahmadabadi M., Naghdi F., Habibi-Parsa M., Haririan I. (2014). Control of superelastic behavior of NiTi wires aided by thermomechanical treatment with reference to three-point bending. J. Mater. Eng. Perform..

[7] Dechkunakorn S., Isarapatanapong R., Anuwongnukroh N., Chiranavanit N., Kajorchaiyakul J., Khantachawana A. (2011). Mechanical properties of several NiTi alloy wires in three-point bending tests. Appl. Mech. Mater..

[8] Phermsang-ngarm P., Charoemratrote C. (2018). Comparison of the load-deflection characteristics of 0.012″ heat-activated, superelastic and bent superelastic nickel titanium wires. Orthod. Waves.

[9] Aghili H., Yasssaei S., Ahmadabadi M.N., Joshan N. (2015). Load deflection characteristics of nickel titanium initial archwires. J. Dent..

[10] Nucera R., Gatto E., Borsellino C., Aceto P., Fabiano F., Matarese G., Perillo L., Cordasco G. (2014). Influence of bracket-slot design on the forces released by superelastic nickel-titanium alignment wires in different deflection configurations. Angle Orthod..

[11] Razali M.F., Mahmud A.S. (2019). Computational study on the effect of contact friction towards deactivation force of superelastic NiTi arch wire in a bracket system. Mater. Res. Express.

[12] Chen H., Han B., Xu T. (2019). Effect of different combinations of bracket, archwire and ligature on resistance to sliding and axial rotational control during the first stage of orthodontic treatment: An in-vitro study. Korean J. Orthod..

[13] Kato M., Namura Y., Yoneyama T., Shimizu N. (2018). Effect of the vertical position of the canine on the frictional/orthodontic force ratio of Ni-Ti archwires during the levelling phase of orthodontic treatment. J. Oral Sci..

[14] Murayama M., Namura Y., Tamura T., Iwai H., Shimizu N. (2013). Relationship between friction force and orthodontic force at the leveling stage using a coated wire. J. Appl. Oral Sci..

[15] Jiang S.-Y., Zhang Y.-Q., Zhao Y.-N., Liu S.-W., Hu L., Zhao C.-Z. (2015). Influence of Ni_4_Ti_3_ precipitates on phase transformation of NiTi shape memory alloy. Trans. Nonferr. Met. Soc. China.

[16] Jiang S.-Y., Zhao Y.-N., Zhang Y.-Q., Hu L., Liang Y.-L. (2013). Effect of solution treatment and aging on microstructural evolution and mechanical behavior of NiTi shape memory alloy. Trans. Nonferr. Met. Soc. China.

[17] Silva J.D., Martins S.C., de Azevedo Lopes N.I., Resende P.D., Santos L.A., Buono V.T.L. (2019). Effects of aging treatments on the fatigue resistance of superelastic NiTi wires. Mater. Sci. Eng. A.

[18] Yi X., Gao W., Wang H., Yao W., Meng X., Gao Z., Cai W., Zhao L. (2018). Dependence of aging parameters on precipitation behavior, martensitic transformation and mechanical properties of the aged Ni-Ti alloy under super high pressure. Mater. Sci. Eng. A.

[19] Dutkiewicz J., Rogal L., Kalita D., Berent K., Kawalko J., Antolak-Dudka A., Durejko T., Czujko T., Cesari E. (2021). Microstructure and mechanical properties of LENS manufactured NiTi shape memory alloy after ageing and during in-situ SEM tensile test. J. Mater. Sci. Metall..

[20] Karimzadeh M., Aboutalebi M.R., Salehi M.T., Abbasi S.M., Morakabati M. (2016). Adjustment of aging temperature for reaching superelasticity in highly Ni-rich Ti-51.5 Ni NiTi shape memory alloy. Mater. Manuf. Process..

[21] Cam G., Kocak M., Dobi D., Heikinheimo L., Siren M. (1997). Fracture behaviour of diffusion bonded bimaterial Ti-Al joints. Sci. Technol. Weld. Join..

[22] Kocak M., Pakdil M., Cam G. (2002). Fracture behaviour of diffusion bonded Ti-alloys with strength mismatch. Sci. Technol. Weld. Join..

[23] (2014). Dentistry—Wires for Use in Orthodontics.

[24] Elkhal Letaief W., Fathallah A., Hassine T., Gamaoun F. (2018). Finite element analysis of hydrogen effects on superelastic NiTi shape memory alloys: Orthodontic application. J. Intell. Mater. Syst. Struct..

[25] Gannoun M., Hellara M.L., Bouby C., Ben Zineb T., Bouraoui T. (2018). Numerical simulation of the force generated by a superelastic NiTi orthodontic archwire during tooth alignment phase: Comparison between different constitutive models. Mater. Res. Express.

[26] Razali M.F., Mahmud A.S., Mokhtar N. (2018). Force delivery of NiTi orthodontic arch wire at different magnitude of deflections and temperatures: A finite element study. J. Mech. Behav. Biomed. Mater..

[27] Naziris K., Piro N.E., Jäger R., Schmidt F., Elkholy F., Lapatki B.G. (2019). Experimental friction and deflection forces of orthodontic leveling archwires in three-bracket model experiments. J. Orofac. Orthop..

[28] Fan Q.C., Zhang Y.H., Wang Y.Y., Sun M.Y., Meng Y.T., Huang S.K., Wen Y.H. (2017). Influences of transformation behavior and precipitates on the deformation behavior of Ni-rich NiTi alloys. Mater. Sci. Eng. A.

[29] Lee J., Shin Y.C. (2019). Effects of composition and post heat treatment on shape memory characteristics and mechanical properties for laser direct deposited nitinol. Lasers Manuf. Mater. Process..

[30] Pu Z., Du D., Zhang D., Li Z., Xue S., Xi R., Wang X., Chang B. (2023). Improvement of tensile superelasticity by aging treatment of NiTi shape memory alloys fabricated by electron beam wire-feed additive manufacturing. J. Mater. Sci. Technol..

[31] Yu H., Qiu Y., Young M.L. (2021). Influence of Ni_4_Ti_3_ precipitate on pseudoelasticity of austenitic NiTi shape memory alloys deformed at high strain rate. Mater. Sci. Eng. A.

[32] Bhardwaj A., Ojha M., Garudapalli A., Gupta A.K. (2021). Microstructural, mechanical and strain hardening behaviour of NiTi alloy subjected to constrained groove pressing and ageing treatment. J. Mater. Process. Technol..

[33] Davim J.P. (2019). Mechanical Behavior of Biomaterials.

[34] Adharapurapu R.R., Jiang F., Vecchio K.S. (2010). Aging effects on hardness and dynamic compressive behavior of Ti–55Ni (at.%) alloy. Mater. Sci. Eng. A.

[35] Benafan O., Garg A., Noebe R.D., Skorpenske H.D., An K., Schell N. (2017). Deformation characteristics of the intermetallic alloy 60NiTi. Intermetallics.

[36] Fernandes F.M.B., Cruz J.M., Magalhães R.C.A. (2015). Comparative study of NiTi orthodontic wires. Mater. Today Proc..

[37] Proffit W., Fields H., Larson B., Sarver D. (2018). Contemporary orthodontics-e-book. Elsevier Health Sciences.

[38] Manazza F., la Rocca S., Nagni M., Chirico L., Cattoni F. (2021). A simplified digital workflow for the prosthetic finishing of implant rehabilitations: A case report. J. Biol. Regul. Homeost. Agents.

[39] D’Orto B., Polizzi E., Nagni M., Tetè G., Capparè P. (2022). Full arch implant-prosthetic rehabilitation in patients with type I diabetes mellitus: Retrospective clinical study with 10 year follow-up. Int. J. Environ. Res. Public Health.

[40] Ciancaglini R., Gherlone E.F., Radaelli G. (1999). Association between loss of occlusal support and symptoms of functional disturbances of the masticatory system. J. Oral Rehabil..

